# Early Occupational Therapy Intervention in the Hospital Discharge after Stroke

**DOI:** 10.3390/ijerph182412877

**Published:** 2021-12-07

**Authors:** Patricia García-Pérez, María del Carmen Rodríguez-Martínez, José Pablo Lara, Carlos de la Cruz-Cosme

**Affiliations:** 1 Faculty of Medicine, University of Málaga, 29010 Málaga, Spain; terapiaocupacional.patricia@gmail.com; 2 Occupational Therapy Department, Hospital Marítimo, Servicio Andaluz de Salud (SAS), 29620 Málaga, Spain; 3 Department of Physiotherapy, Faculty of Health Sciences, University of Málaga, 29071 Málaga, Spain; 4 Brain Health Unit, CIMES, 29010 Málaga, Spain; 5 Malaga Biomedical Research Institute (IBIMA), 29010 Málaga, Spain; carlosdelacruzcosme@gmail.com; 6 Neurology Department, Virgen de la Victoria University Hospital, 29010 Málaga, Spain

**Keywords:** stroke, patient discharge, occupational therapy, rehabilitation, caregivers, prevention, universal health coverage

## Abstract

Stroke is the leading cause of acquired disability in adults which is a cerebrovascular disease of great impact in health and social terms, not only due to its prevalence and incidence but also because of its significant consequences in terms of patient dependence and its consequent impact on the patient and family lives. The general objective of this study is to determine whether an early occupational therapy intervention at hospital discharge after suffering a stroke has a positive effect on the functional independence of the patient three months after discharge—the patient’s level of independence being the main focus of this research. Data will be collected on readmissions to hospitals, mortality, returns to work and returns to driving, as well as an economic health analysis. This is a prospective, randomized, controlled clinical trial. The sample size will be made up of 60 patients who suffered a stroke and were discharged from the neurology unit of a second-level hospital in west Malaga (Spain), who were then referred to the rehabilitation service by the joint decision of the neurology and rehabilitation department. The patients and caregivers assigned to the experimental group were included in an early occupational therapy intervention program and compared with a control group that receives usual care.

## 1. Introduction

Activity limitations are difficulties an individual may have in executing activities. In order to reduce activity limitations and improve functional independence, the occupational therapist can intervene through personal assistance, rehabilitation therapy and the use of assistive devices. Preventive rehabilitation and the prevention of participation restrictions are also a part of the intervention that become really important after suffering a stroke, and especially before returning home from hospital [1].

By stroke or cerebrovascular accident (CVA) we refer to those sudden circulatory system episodes that compromise the stability of an affected brain area, either permanently or temporarily. As a result, we will use the defined terms to refer to cerebral infarction, as well as intracerebral and subarachnoid hemorrhages [2]. Stroke is a cerebrovascular disease with great health and social impacts due to its high incidence and prevalence, and for being the leading cause of acquired disability in adults in developed countries. Its incidence in Spain is 187.4/100,000 inhabitants/year. It is a great burden, not only from a health point of view, but also from a personal and family point of view, due to its impact on the lives of the people who suffered it and their caregivers [3,4]. It is also one of the main causes of hospital stay and death in Spain. The relevance of a stroke is given by its mortality data, as well as the dependence of the survivors. Studies show that after the first six months, significant clinical and functional stabilization is achieved. Among the survivors, 30–40% will present some serious sequelae, and up to 60% of users will present minor sequelae or have no sequelae, and only 6% of cases with severe initial paralysis will fully regain mobility. This means that more than 90% will present some activity limitation which could be trained by the figure of an occupational therapist in order to pursue activity execution success and therefore reduce their dependence and improve their quality of life. These data demonstrate the importance of rehabilitation to facilitate relearning for these patients, which has been shown in previous studies to be useful in the recovery of the user in terms of functional autonomy as well as increasing the frequency of returning home and reducing the hospital stay [5,6,7,8]. Likewise, a caregiver-mediated program has been shown to have the potential to improve the body function, activity, and participation outcomes of people with stroke. In addition to this, caregivers participate more actively in the rehabilitation process, which can increase feelings of empowerment, reducing burden levels on the caregiver, and facilitating the transition from hospital to home [9,10,11].

Generally, the initial assistance for stroke intervention begins with a call to the services of 061 or 112 and the arrival at the Emergency Department. Subsequently, the user is admitted to the Stroke Unit and once stabilized, they are transferred to the neurology ward. The importance of rehabilitation in the acute phase is given by the existence of a therapeutic window during which interventions can actually modify the course of the disease and achieve neuronal reactivation. This improvement may be due to the existence of a penumbra area on the periphery of the ischemic area, the damage of which is potentially reversible, although it can only be achieved for a short and variable time of up to 24 h if the reperfusion of said tissue is achieved, as well as the resolution of diaschisis (transsynaptic failure of neurons connected to the damaged area). However, there is a brain reorganization that can be modulated by rehabilitation techniques through the phenomenon of neuronal plasticity, which determines the importance of an efficient and early multidisciplinary intervention [9,12].

The role of the occupational therapist has its place at all levels of stroke intervention, both in direct action with the user, as in counseling and family or social support. Occupational intervention is initially directed at sensory–motor and perceptual–cognitive performance skills, as well as re-education and training in the basic and instrumental activities of daily living (ADL); subsequently, intervention more oriented towards the social and labor integration of the person is proposed [13]. A systematic review of the scientific literature on this topic was conducted by the authors, showing that occupational therapy intervention during hospital discharge for people post-stroke could be beneficial in terms of the patient’s functional improvement and caregiver burden [14,15,16,17].

Occupational therapy (OT) intervenes on function, using specific procedures and activities to develop, maintain, improve and/or recover the performance of functions and activities necessary in daily life, compensate for dysfunctions and/or promote health and well-being. According to the framework for the practice of occupational therapy, we classified this function in areas of occupation (basic and instrumental ADL, health management, rest and sleep, education, work, play, leisure and social participation) and performance skills (motor skills and praxis, sensory–perceptual skills, emotional regulation skills, cognitive skills, and social and communication skills). Consequently, the initial objective of an OT intervention plan is to improve the user independence in performing ADL by reducing activity limitations and training them to achieve the highest possible level of autonomy, therefore promoting their well-being and quality of life [13,18].

According to a study published in 2015, the prognosis of stroke in Spain has changed in recent years. Both survival and functional outcomes have improved as a result of the introduction of a new model of care. This new model, based on stroke units, early reperfusion therapies, physiotherapy and secondary prevention, has been promoted by the stroke strategy of the National Health Service of Spain in coordination with regional health authorities and health professionals. However, this model does not take into account the OT intervention in the discharge process, as other countries do with positive results in functional independence, among others [14,15,16,17,19].

Finally, it should be remembered that this pathology also has a relevant economic impact on the national direct health cost, plus those derived from its employment and socio-family repercussions. According to a recently published study, the cost of patients admitted to stroke units in Spain is EUR 27,711 per patient/year. More than two thirds are social costs, mainly informal care [20,21,22,23].

The general objective of this study is to determine whether occupational therapy (OT) intervention together with the usual care in the process of hospital discharge after suffering a stroke has a positive effect on the functional independence of the patient, comparing it with the control group that will have regular care and rehabilitation assistance.

We designed a four-week OT intervention program with the support of the main caregiver of patients who had suffered a stroke with the aim of improving the functional result and facilitating the return home by providing the caregiver with knowledge of specific care and neurorehabilitation, which could also lead to a reduction in costs to the health system.

The specific objectives of the study are:

- Primary outcome: functional independence and support needs in activities of daily living;
- Secondary outcomes: improvement in sensory–motor skills, perceptual–cognitive skills, communication skills, quality of life, levels of anxiety and depression, and coping strategies and caregiver burden. At the same time, data on readmissions to the hospital, mortality, age, sex, etiology, race, income, side of hemiparesis, vascular territory and volume of the lesion, reperfusion treatment received, caregiver support, medical treatment at discharge, return to work, and return to driving are collected, as well as a direct economic analysis.

The hypothesis of this study is that an early occupational therapy intervention in the process of hospital discharge post-stroke has a positive effect on the functional independence of the patient. Therefore, it can lead to improvements in sensory–motor skills, perceptual–cognitive skills, communication skills, quality of life, levels of anxiety and depression and also reduce the caregiver burden.

## 2. Relevance

The early supported discharge model (ESD) for rehabilitation was introduced in the late 1990s and includes an interdisciplinary team with adequate resources that coordinates discharge and plans, supervises and continues rehabilitation in the home setting [24].

Based on randomized controlled clinical trials, there is evidence that early discharge with follow up from the hospital (ESD) and continuous rehabilitation at home has beneficial effects on stroke victims; however, the effects of the ESD service in routine clinical practice in Spain have not been investigated [25,26]. Similarly, there is a lack of published data regarding the impact of occupational therapy in the hospital discharge process together with conventional care and rehabilitation in terms of the functional results of the patient, caregiver burden, and costs of medical care during the first six months after the stroke [27].

There are at least three important perspectives to take into account in any assessment of actions taken on stroke sequelae: personal perspective, social perspective, and public health. From a personal perspective, returning home after a hospital stay due to stroke and being able to participate in the rehabilitation plan and goal setting results in patient empowerment and greater satisfaction, improving the mood and promoting well-being. From a social perspective, it can help the family feel less stressed as support is provided at home, it can reduce the risk of readmission and stress-induced symptoms in relatives. It is also an opportunity to improve the caregiver’s quality-of-life and reduce their risk of illness, as family members often report feeling a heavy burden as caregivers. From a public health perspective, the possibility of ensuring a safe discharge with the support of occupational therapy can lead to a reduction in costs [12].

It is important to consider the involvement of the family in the rehabilitation process, as well as their knowledge regarding the disease and specific care. Their involvement can provide a stimulating and safe environment at home that reduces the risk of pressure injuries, muscle or soft tissue shortening, dislocations, subluxation, etc., in patients with motor impairment. The treatment provided in the current public health system is insufficient, which can be worrying due to the limited recovery after stroke and the difficulty in community reintegration. That is why certain variables are of special interest when trying to weigh the effectiveness of this type of action, such as motor improvement and the importance of the functional mobility of the upper extremities, which can contribute to a better quality of life for stroke survivors [27,28].

Moreover, there are no studies in our environment that demonstrate whether discharge with OT support is effective and profitable, whether it increases the safety of patients and their families, or whether patients perceive less anxiety and depression, higher levels of functional independence and better quality of life.

## 3. Materials and Methods

### 3.1. Type and General Design of the Study

This will be a prospective, randomized, controlled clinical trial. The sample size was made up of 60 patients who will be divided into two groups: the control group, with 30 users, and the experimental group, with another 30 users. The population to be represented will be made up of patients who have been discharged from the neurology service of the Virgen de la Victoria University Hospital (Malaga), and who, after discharge, were referred to the rehabilitation service due to a joint decision of the neurology and rehabilitation departments, but who are not subsidiaries of the inpatient unit for intensive rehabilitation at the Marítimo Hospital.

**Figure 1:**
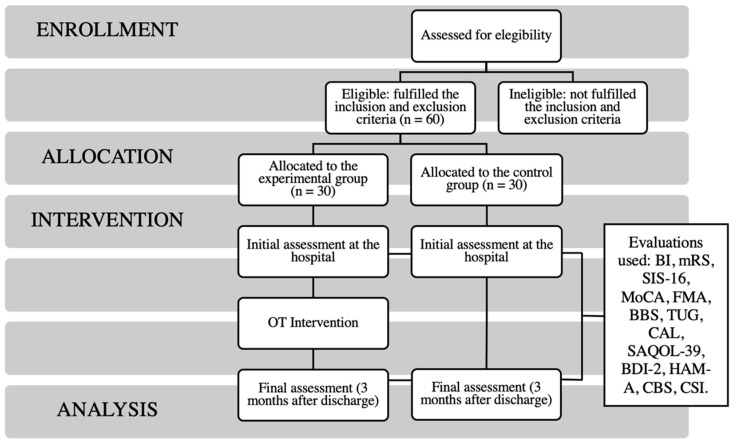
Protocol design implemented in the study.

Users who are referred to rehabilitation and are subsidiaries of the inpatient unit receive daily occupational therapy and physical therapy interventions for a maximum period of three months. However, patients who are referred to the rehabilitation service as an outpatient are directly taken home after discharge and are subsequently called in for rehabilitation. The period that elapses from when they are discharged until they are called to begin their rehabilitation varies according to the workload of the service, and may even take three months after discharge. Referral to external or intensive rehabilitation is made based on the rehabilitation potential, which takes into account certain criteria such as age, clinical situation and vascular injury at the time of discharge, social support, motivation and observed recovery during admission. Patients and caregivers assigned to the experimental group are included in an early occupational therapy intervention program and compared with a control group that receives usual care and rehabilitation. Figure 1 shows a flow chart of the study design.

This protocol study has been registered at clinicaltrials.gov (accessed on 26 October 2021) with the registration number: NCT04835363 (registered on 25 March 2021). 

### 3.2. Selection and Sample Size

The sample size ″n″ was calculated to represent the total population of patients who suffer a stroke admitted to the Virgen de la Victoria University Hospital in Malaga, and who after the neurological intervention are left with sequelae susceptible to rehabilitation but who do not acquire a place as an inpatient in the rehabilitation unit at the Marítimo Hospital (a total of 800 admissions for strokes in the Hospital per year was calculated).

Assuming a Barthel index as the main variable of the study and relying on the mean values and standard deviation of the literature, with 30 patients in each group, a difference detected between the two groups of the 25% will be considered significant. For this, we are assuming a level of significance of 95% and a power of 80%.

Therefore, two groups will be formed: an intervention group of 30 patients who will be included in the early occupational therapy program in addition to the rehabilitation that they would receive regardless of this study, and a control group of 30 patients who will receive the usual care and rehabilitation. Assignment to the control or intervention group will be done randomly.

### 3.3. Inclusion and Exclusion Criteria

All patients will need a diagnostic confirmation of stroke with single or multiple vascular lesions that occurred during the same time period, demonstrated by neuroimaging tests (CT or MRI), be over 18 years of age, and must live within a maximum of 30 min away from the hospital center, with >2 to <26 points on the National Institute of Health (NIHSS) scale, 45–100 points on the Barthel Index (BI) on the second day of the stroke (with BI 100, the patient can be included if the Montreal Cognitive Assessment is <26) and must present some motor deficit that makes it difficult to carry out their ADL. Inclusion in the study occurred prior to hospital discharge.

Exclusion criteria were: NIHSS > 26; BI < 45; and life expectancy < 1 year. People who have previously suffered a stroke, dementia or other types of illnesses associated with dementia and other concomitants neurological, psychiatric or medical illnesses (for example, severe epilepsy, head trauma, schizophrenia, COPD, severe or unstable heart disease, sleep apnea) that could alter cognitive function, as well as people who cannot understand Spanish or English, will also be excluded.

Variables that will be taken into account are: age, sex, etiology, race, income, side of hemiparesis, vascular territory and volume of the lesion, reperfusion treatment received, caregiver support and medical treatment at discharge.

### 3.4. Assessments to Be Used in the Study

Assessments used in the study are described in the following paragraphs and Table 1.

Montreal Cognitive Assessment (MoCA) was proposed as a tool that promises good sensitivity to deficits that result from stroke and vascular cognitive impairment. The MoCA includes sections on visuospatial/executive functions, naming, attention, language, abstraction, memory, and orientation. It is scored out of 30 (extra point for <13 years of education) and the recommended “normal” limit is ≥26 [29,30].

The Modified Rankin Scale (mRS) is used to describe disability in general. The scale ranges from 0 to 6, from perfect health without symptoms to death [31].

The Fugl–Meyer sensory motor assessment (FMA) evaluates the upper limb (maximum score of 66 corresponding to normal motor function) and lower extremity (maximum score of 34). It also evaluates sensitivity, passive range of motion and pain during passive joint movements [32].

Timed up and go (TUG) assesses basic mobility, counting the time required for a person to get up from a standardized chair, walk a distance of three meters, turn, return to the chair, and sit down again. A shorter time indicates better performance. It has been shown to be reliable and valid in this group of patients [33].

The Berg Balance Scale (BBS) assesses functional balance. Performance on this test is rated from 0 (cannot perform) to 4 (normal performance) on 14 different tasks, including the ability to sit, stand, reach, lean, roll over and step. The maximum score on the BBS is 56. A higher score indicates better balance skills [34].

The Barthel Index (BI) measures the extent to which someone can function independently during basic ADL, i.e., feeding, bathing, grooming, dressing, bowel control, bladder control, grooming, chair transfer, ambulation and stair climbing. Each performance item is rated on this scale with a given number of points assigned to each level. The modified version with 0–100 was used in this study, where a lower score indicates higher dependence [35].

Stroke and aphasia quality of life Scale-39 (SAQOL-39) was derived from the Stroke-Specific Quality of Life Scale including four additional items specifically targeting aphasia patients. It has four domains: physical, psychosocial, communication and energy [36].

The Stroke Impact Scale-16 (SIS-16) has a usefulness is similar to that of the Barthel Index, although it is more sensitive than the latter to discriminate between patients with mild disabilities. It was especially designed for patients who have suffered strokes, but it is also applicable to dementia. It is a reduced version of the Stroke Impact Scale version 3.0. The information can be obtained from the same patient, or from the main caregiver [37,38,39].

The Communicative Activity Log (CAL) scale allows obtaining information on communication skills in activities of daily life referring to comprehensive and expressive aspects of language. It is made up of 36 items (Likert-type scale, 0–6) that assess both the quality (items 1–18) and the quantity of the patient’s communication (items 19–36). It can be applied by self-administration or hetero-administration. The performance is obtained through the sum of the scores in each item [40].

The Beck Depression Inventory (BDI-2) is a self-administered questionnaire that consists of 21 multiple-choice questions. It is one of the most commonly used instruments to measure the severity of depression [41].

The Hamilton anxiety scale (HAM-A) assesses the severity of anxiety globally in patients who meet criteria for anxiety or depression. Furthermore, this instrument is useful for monitoring the response to treatment. It is made up of 14 items, 13 of which refer to anxious signs and symptoms and the last one that assesses the patient’s behavior during the interview [42].

The main purpose of the Inventory of Coping Strategies (CSI), as it describes, is to find the type of situations that cause problems for people in their daily lives and how they deal with these problems. This inventory collects two types of information: one, qualitative, where the person describes the stressful situation; and another, quantitative, where the frequency of use of certain coping strategies according to a Likert scale is answered, as well as the degree of perceived efficacy in coping [43].

The Caregiver Burden Scale (CBS) is a questionnaire with 22 questions (answered in writing by the caregiver) about the burden of the caregiver health aspects, the feeling of psychological well-being, relationships, the social network, physical workload and environmental aspects that can be important. When the scale was developed, factor analysis was used to obtain five indices: general tension (8 questions); disappointment (5 questions); isolation (3 questions); emotional involvement (3 questions); and environment (3 questions). Items are scored from 0 to 3 (not at all, hardly, somewhat and definitely, respectively) with a maximum score 66 [44].

The research includes data on comorbidity, hospital readmission and mortality, as well as questions and answers about the subjective experience of the OT intervention. Data will also be included on the satisfaction with the information provided on stroke and where to obtain support, as well as the opinions of patients and/or relatives on the adequacy or not of home care assistance and support. Table 2 describes qualitative and secondary data included in the research. 

The evaluations and interviews will be carried out with a strategic sample of 60 patients and caregivers before discharge (after informed consent) and three months after the neurological event in order to describe the subjective expectations and experiences of both the caregivers and patients regarding the discharge process and rehabilitation received. For this purpose, a structured interview guide will be used. The interviews will be analyzed with quantitative and qualitative content analysis.

Finally, an economic analysis will be carried out taking into account the data on expenses related to healthcare (days of stay in the hospital for hospital readmission if it occurs and the number of rehabilitation sessions) and family expenses (estimated expense of each family in the first three months after the stroke), as well as other expenses that may have arisen. Data on family spending will be collected in the semi-structured interview.

### 3.5. Schedule and Data Collection

The rehabilitation and neurology departments will be responsible for referring patients to the occupational therapist and including them in the study. Before beginning rehabilitation, the occupational therapist and their family explore the needs and wishes of the patient and all together decide on individual goals for the intervention period.

Occupational therapy is a client-centered health profession concerned with promoting health and well-being through occupation. The primary goal of occupational therapy is to enable people to participate in the activities of everyday life. Occupational therapists achieve this outcome by working with people and communities to enhance their ability to engage in the occupations they want to, need to, or are expected to do, or by modifying the occupation or the environment to better support their occupational engagement.

Occupational therapy intervention is directed towards: providing comprehensive assessment, improving functional levels, facilitating discharge from hospital, organizing a safe home environment and ensuring continued rehabilitation at home with caregiver support if available. Occupational therapy intervention is client-centered; therefore, each intervention will be specifically designed for each patient. Examples of occupational therapy intervention may include: planning and facilitating discharge from hospital, assessment and training of activities of daily living (ADL), physical rehabilitation, cognitive rehabilitation, recommending equipment to enable ongoing independence following discharge from hospital and training in use of equipment to achieve independence, the adaptation of the home environment, providing advice to the patient and carers, promotion of health and coping strategies, mobilization of joints, building up strength, dexterity and work tolerance [45].

The intervention is based on who the user is: their context, their history, their family members, their individual strengths and weaknesses. Individualized objectives will be established, taking into account potentialities and limitations. Examples of goals might be: being able to wash independently, hang up clothes or ride the bus, or how to manage bills. OT intervention may involve training in different activities or thinking of different ways to adapt to difficult situations in order to achieve successful activity execution. For example, for some patients, the intervention may consist of training an ADL in which they present a limitation with the support of the occupational therapist in order to enhance their sense of security during the performance of this activity. The data will be collected through note taking and analyzed with qualitative content analysis.

The designed OT intervention includes:

- Before discharge: Two visits to the hospital.
- Post-discharge: A post-discharge home visit, telephone follow up, a home visit one month later, and a final evaluation three months after discharge.

This early OT intervention will be carried out independently of the usual care of the rehabilitation service and that of the Hospital Marítimo in Torremolinos.

### 3.6. Temporary Planning

The field project will begin in 2021 and is estimated to last approximately one year. The temporary planning is shown in Table 3. 

### 3.7. Data Analysis Section

The following analysis model will be followed: to study the differences that may exist between one group and another, before and after, a Student’s *t*-test will be performed; to check how some variables influence each other, a multivariate analysis can be carried out and, finally, to check the mean and normal distribution of the variables, a descriptive statistic will be made. It is important to note that each patient is in their own control and, therefore, it is necessary to measure the percentage of change in each patient in the different evaluations (intragroup study).

The program to be used for data analysis will be IBM^®^ SPSS^®^ Statistics 26.0, (IBM, Chicago, IL, USA). 

## 4. Discussion

Due to the great number of stroke patients that go into hospital per year, it is expected to achieve the total sample size and complete allocation and enrolment tasks by the first year of RCT. Many studies have shown that OT seems to be an effective post-stroke rehabilitation approach which can induce meaningful functional improvements. Moreover, studies show the impact of instrumental successful execution activities of daily living on hospital readmission, suggesting limitations on these activities are key predictors of 30-day readmissions [21,22,23].

Regarding the possible limitations of this study, it is worth highlighting the biases that may derive from the adherence to treatment of the users and their caregivers as well as the behavior and involvement of the family member in the process that could lead to differences in the results. The importance of the word of the patient and the caregiver should be highlighted due to the telephone intervention process, since it could lead to differences in the instructions provided by the occupational therapist and, therefore, in the treatment and the final result.

Furthermore, an important limitation could be the heterogeneity of the sample, since we are generally speaking of patients who have had a stroke at any location to any extent and volume. The random distribution of patients may have as a consequence that the two groups have important significant differences due to the severity of their injuries or their potential for recovery. Additionally, due to the heterogeneity of the rehabilitation process that each patient receives from hospital, it is understood that, on the total sample size, certain patients will consequently receive a greater number of sessions than others, and probably some patients will not receive any sessions. This is why it could also be interesting to study the care received by each user 6 months after discharge, as well as their level of independence.

## 5. Conclusions

Although the scientific evidence published on this topic has increased in recent years, further well-designed randomized controlled trials are necessary to confirm the benefits of associations between different treatments in a patient’s functional outcome [46].

The development of this study will contribute to a better adherence to the treatment of users and improve the quality of life of both users and their families. Likewise, the implementation of this protocol in Spain can be very significant in clinical practice, since in other countries, the figure of the occupational therapist is already very relevant in the discharge process after stroke, while in Spain, there is still no evidence of this type of intervention. Finally, a last novelty that this protocol provides is that it is intended to carry out a holistic program that considers physical, cognitive and psychosocial aspects, considering the person as the key point of their recovery. 

## Figures and Tables

**Table 1:** List of objectives and evaluations.

**Patient Evaluations**	
Level of functional independence	BI mRS SIS-16
Perceptual–cognitive skills	MoCA
Sensory–motor skills	FMA BBS TUG
Communication skills	CAL
Quality of life	SAQOL-39
Levels of anxiety and/or depression	BDI-2 HAM-A
**Caregiver Evaluations**	
Caregiver burden	CBS
Coping strategies	CSI

**Table 2:** List of other results and qualitative data.

**Related to Health and Independence**
Comorbidity Hospital readmission Subjective experience of the OT intervention Satisfaction with information provided on stroke and where to obtain support Opinion of patients and/or relatives on the adequacy of home care assistance and support ADL execution success and goals achieved Return to work Return to driving
**Economics Data**
Expenses related to health care Family expenses

**Table 3:** Temporary planning.

Day 1: Hospital (1 h) Initial interview and evaluation.
Day 2: Hospital (1 h) Information about the ACV. Postural care.
Day 3: Housing (2 h) Study of the environment, recommendations and support products and establish individualized objectives.
Day 4–day 29: PHONE FOLLOW-UP. Initiation of rehabilitation by the caregiver (individual objectives).
Day 30: Housing (2 h). Final assessment of AVD at home.
At 3 months: Housing (2 h) Final evaluation.

## Data Availability

Not applicable.
